# Insecticidal and Feeding Deterrent Effects of Fraxinellone from *Dictamnus dasycarpus* against Four Major Pests

**DOI:** 10.3390/molecules18032754

**Published:** 2013-03-01

**Authors:** Min Lü, Wenjun Wu, Huixia Liu

**Affiliations:** Institute of Pesticide Science, College of Plant Protection, Northwest A&F University, Yangling 712100, Shaanxi, China; E-Mail: Wuwenjun-1@163.com

**Keywords:** fraxinellone, antifeedant activity, ovicidal action, development change

## Abstract

Fraxinellone, a well-known and significant naturally occurring compound isolated from Meliaceae and Rutaceae spp. has been widely used as a drug for the treatment of tumors. On the other hand, fraxinellone exhibited a variety of insecticidal activities including feeding-deterrent activity, inhibition of growth, and larvicidal activity. The present study focused on the antifeedant and larvicidal activities of fraxinellone against the larvae of Lepidoptera, including *Mythimna separata*, *Agrotis ypsilon*, *Plutella xylostella*, and one kind of sanitary pest, *Culux pipiens pallens*. Meanwhile, the ovicidal activities and the effects of fraxinellone on the larval development of *M. separata* were also observed. The LC_50_ values of fraxinellone against 3rd instar larvae of *M. separata*, 2nd instar larvae of *P. xylostella* and 4th instar larvae of *C. pipiens pallens* were 15.95/6.43/3.60 × 10^−2^ mg mL^−1^, and its AFC_50_ values against 5th instar larvae of *M. separata*, 2nd instar larvae of *P. xylostella* and 2nd instar larvae of *A. ypsilon* were 10.73/7.93/12.58 mg mL^−1^, respectively. Compared with the control group, fraxinellone obviously inhibited the pupation rate and the growth of *M. separata*. Once *M. separata* was treated with fraxinellone at concentrations of 5.0, 10.0, and 20.0 mg mL^−1^, respectively, the stages from the larvae to adulthood and the egg hatching duration were prolonged to 1/2/3, and 4/3/4 days, respectively. Additionally, fraxinellone strongly inhibited the development rate and the egg hatch proportion of *M. separata*.

## 1. Introduction

Plant secondary metabolites result from the interaction between plants and environment (living and non-living) during the long period of evolution in plants and play an important role in protecting plants from herbivores. Higher plants contribute significantly to this success as sources for highly active agents, e.g., pyrethroids, nicotine, avermectins and rotenone, *etc.*, widely used in pest management. More than 2,400 plant species have been found to control crop pests. Currently, the discovery of new insecticidal compounds from plant secondary metabolites and subsequently using them as the lead-compounds for further modification has been one of the important approaches for the research and development of new pesticides [1,2].

**Figure 1:**
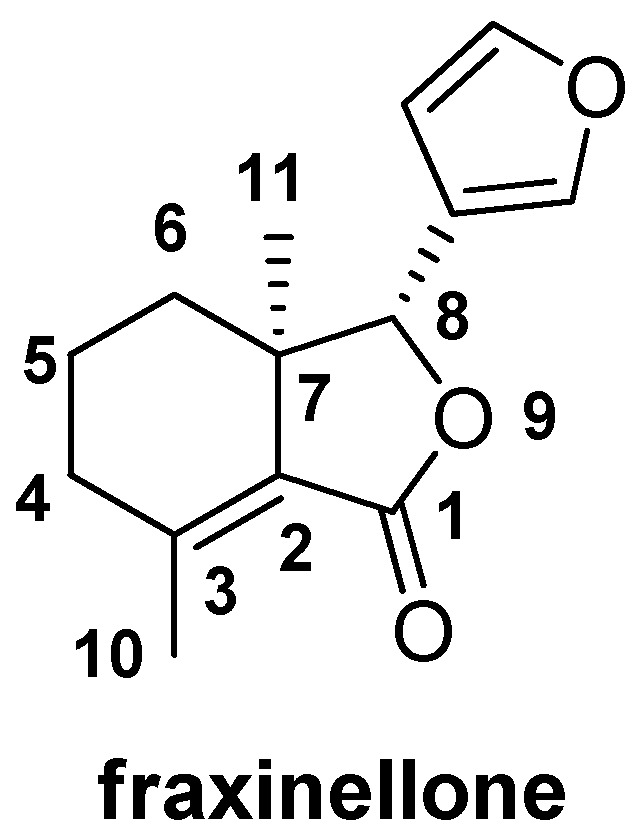
The chemical structure of fraxinellone.

Fraxinellone (Figure 1), a well-known and significant naturally occurring degraded limonoid, has been successfully isolated and identified from many species of plants in the Meliaceae and Rutaceae families, including *Dictamnus angustifolius* [3,4], *Fagaropsis glabra* [5], *Melia azadarach* [6,7], *Raulinoa echinata* [8,9], and *D. dasycarpus* [10,11]. For example, the extracts of *Azadirachta indica* (neem), *A. excelsa* (sentang), *Melia volkensii*, *M. azedarach* (Chinaberry) and *Trichilia americana*, (all belonging to the family Meliaceae) proved to be strong growth inhibitors, contact toxins and significant feeding deterrents towards two noctuid caterpillars, *Trichoplusia ni* and *Pseudaletia unipuncta* [12]. In China, the dried root bark of *D. dasycarpus* Turcz., family Rutaceae, exhibited antifertility, antiplatelet aggregation and vascular relaxing activities [13,14], and has been widely used for the treatment of cough, rheumatism, skin diseases, and so on [15]. Furthermore, fraxinellone, besides its use for the preparation of potent anticancer drugs, has also received much research attention for its interesting insecticidal and fungicidal activities [16]. In 1997, Okamura *et al*., reported the total synthesis of fraxinellone [17]. More recently, Xu and co-workers have synthesized fraxinellone-based ester/hydrazone derivatives, and found some compounds exhibited more pronounced insecticidal activity as compared with toosendanin [18,19].

Lepidoptera are the most diverse pest insect order. There is hardly a cultivated plant that is not attacked by at least one lepidopteran pest. Insect antifeedant activities of fraxinellone have been exploited against *Spodoptera littoralis* [6], *S. exigua* [10] and armyworm *Mythimna separata* (all belonging to Lepidoptera: Noctuidae) [20,21]. Liu *et al*. [10] found that it displayed significant feeding deterrence activity against two stored-product insects such as *Tribolium castaneum* Herbst (Coleoptera: Tenebrionidae) and *Sitophilus zeamais* Motsch (Coleoptera: Curculionidae). Against adults and larvae of *T. castaneum* and adults of *S. zeamais*, the EC_50_ values of fraxinellone were 36.4, 29.1, and 71.2 ppm, respectively. Similarly, the growth rates and food consumption of adults and larvae of *T. castaneum*, Asian corn borer, *Ostrinia furnacalis* (Lepidoptera: Crambidae) [22] and tobacco budworm, *Heliothis virescens* (Lepidoptera: Noctuidae) [23] were also obviously inhibited by fraxinellone.

After treatment with fraxinellone, the poisoned insects exhibited typical stomach poisoned symptoms (e.g., vomiting and diarrhea), and fraxinellone showed delayed insecticidal activity against *M. separata*. Histopathology research demonstrated that the organelles of *M. separata* midgut were obviously changed after fraxinellone treatment [24]. Encouraged by the above-mentioned interesting results, herein we wanted to further study the antifeedant activities and toxicity of fraxinellone against the larvae of lepidoptera, such as *M. separata*, *Agrotis ypsilon* (Lepidoptera: Noctuidae), and *Plutella xylostella* (Lepidoptera: Plutellidae) and one kind of sanitary pest *Culuxpipiens pallens* (Diptera: Culicidae)*.* Meanwhile, the ovicidal activities and the influence of fraxinellone on the larval development of *M. separata* were also investigated.

## 2. Results and Discussion

### 2.1. Antifeeding and Insecticidal Activities

Antifeedants are described as substances that reduce feeding by an insect and are found among all of the major classes of secondary metabolites: alkaloids, phenolics and terpenoids, with the latter comprising the most potent and diverse forms of antifeedants. In this research, fraxinellone exhibited obvious feeding deterrent activity against most kinds of Lepidopteran larvae (Table 1), such as *M. separata*, *A. ypsilon*, and *P. xylostella*,. Besides that, fraxinellone also possessed toxicity against the larvae of *P. xylostella*, *M. separata* and *C. pipiens pallens.*

**Table 1:** Feeding deterrent and toxic effects of fraxinellone against different pests.

Larvae		AFC_50_^a^ (mg mL^−1^) (95% confidence interval values)	Toxicity curve	Correlation efficient (*r*)	LC_50_^b^ (mg mL^−1^) (95% confidence interval values)	Toxicity curve	Correlation efficient (*r*)
*M. separata*	5th-instar	10.73 (8.7571–13.1406)	y = 2.5268 + 2.4000x	0.9756	/	/	/
	3rd-instar	/	/	/	15.95 (13.459–17.492)	y = 4.4509 + 3.2375x	0.9878
*P. xylostella* (2nd-instar)		7.93 (5.9175–10.6201)	y = 3.1543 + 2.0528x	0.9817	6.43 (4.7050–8.7876)	y = 3.6399 + 1.6829x	0.9901
*A. ypsilon* (2nd-instar)		12.58 (7.6630–20.6561)	y = 3.3786 + 1.4743x	0.9816	/	/	/
*C. pipiens pallens* (4th-instar)		/	/	/	3.60 × 10^−2^ (3.0–4.3) × 10^−2^	y = −1.4386 + 4.1389x	0.9347

^a^ After treatment for 24 h. ^b^ After treatment for 48 h, whereas to *C. pipiens pallens* for 24 h. “/” means not tested.

### 2.2. Egg Hatching Inhibition of M. separata

**Figure 2:**
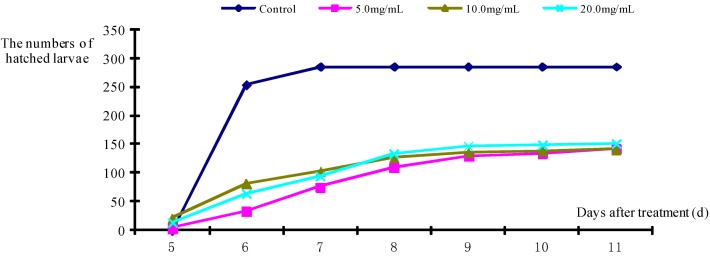
The numbere of hatched larva of *M. separata* caused by different concentrations of fraxinellone along with time.

Fraxinellone exhibited strong egg hatching inhibition ability. For example, after treatment with fraxinellone (Table 2 and Figure 2), the hatching durations of *M. separata* were all delayed to 3–4 days, and a few hatched eggs grew up to become 2nd instar larvae. The effects of different concentrations of fraxinellone solutions on egg hatching inhibition were inconspicuous. For example, after treatment for 8 days (Figure 2), the hatching rate of the control group was 95.65%, whereas the hatching rates of the treated groups were only 26.79%, 38.20% and 30.82% at the concentrations of 5.0, 10.0 and 20.0 mg mL^−1^, respectively. Otherwise, the final hatching ratio were nearly the half of the original quantities of egg, and the egg hatching rates of the treated groups were all decreased by more than 50% when compared with that of the control group. Meanwhile, part of the hatched larvae in the treated groups could not move away from the eggshells, and they ultimately died. Some eggs exhibited embryonic developmental evidence, but they could not hatch before death.

**Table 2:** Effect of fraxinellone on egg hatching rates and hatched-larvae development of *M. separate*.

Treatment Concentration (mg mL^−^^1^)	The number of egg	The number of 2nd instar larva	Hatch duration (day) ^a^	Egg hatching rate (%)	Hatch inhibition rate (%)	Development rate (%) ^b^
Control	299	240	7	95.65	/	83.92
5.0	280	53	11	50.71	46.98	26.06
10.0	267	19	10	52.81	44.79	13.48
20.0	305	26	11	49.51	48.24	14.57

^a^ There were no hatched larvae appeared after the date. ^b^ All the hatched larvae grew up to 2nd instar.

As we all know, the eggshell is the first barrier which protects the embryo from xenobiotics. During the egg hatching, yolk protein acts as a nutritional reserve of amino acids, lipids, hydrocarbons, and vitamins, *etc.* for the embryo, and the synthesis of yolk proteins is regulated by endogenic hormones [25]. Therefore, we deduced that the mechanism of egg hatching inhibition may be the result of destroyed eggshells, changed yolk protein content or reduced ratios of endogenic hormones. At present, related research is being carried out in our group to confirm the above-mentioned hypotheses.

### 2.3. Effect on Larval Development of *M. separata*

**Figure 3:**
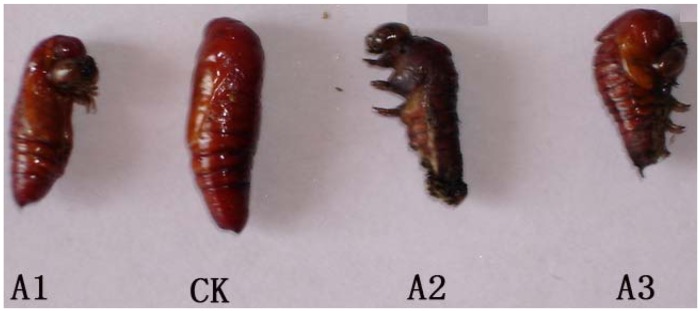
Effect of fraxinellone on *M. separata* pupation (**A1**, **A2** and **A3** were abnormal pupae after treated by fraxinellone; **CK**: blank control group).

As depicted in Table 3, after treatment with fraxinellone for 2 h, the 5th instar larvae of *M. separata* only fed on a few of the treated wheat leaves, and the antifeeding rates were all larger than 60%. The development durations from 5th to 6th instar of *M. separata* were prolonged 1, 2 and 3 days at the concentrations of 5.0, 10.0 and 20.0 mg mL^−1^, respectively. Meanwhile, fraxinellone inhibited the growth of the larvae, *e.g.*, the development inhibition ratios at 10.0 and 20.0 mg mL^−1^ were more than 30%. The pupation ratio and eclosion ratio all declined with increased concentrations of fraxinellone. Additionally, fraxinellone produced the emergence of abnormal pupae, *i.e.*, at 10.0 and 20.0 mg mL^−1^, the percentages of abnormal pupae were 12.5 and 30.0%, respectively (Figure 3).

**Table 3:** Effect of fraxinellone on *M. separata* feeding and larval development.

Treated Concentration (mg mL^−1^)	Mean larval weight (mg)	Antifeeding rate (%)	Mean pupal weight (mg)	Development duration ^a^ (days)	72 h mortality (%)	Corrected abnormal mortality (%)	Development inhibition rate (%)
Control	35.4	/	186.4	7.50 ± 0.24	0	/	/
5.0	36.1	66.6	186.1	8.85 ± 0.48	0	50.00	12.19
10.0	34.6	61.3	211.2	9.75 ± 0.78	16.7	66.63	36.59
20.0	36.4	80.6	195.5	10.15 ± 0.63	23.3	70.88	30.07

^a^ The larvae of control group grew up to the 6th instar.

Isolated from the Meliaceae and Rutaceae families, most limonoids, e.g., azadirachtin and toosendanin, possess antifeedant, toxic or growth control properties against different species of insects. For example, the leaf extracts of Argentine *Melia azedarch* L. [26] and meliternatin, isolated from *Melicope subunifoliolata* (Rutaceae) [27], showed larvicidal and feeding deterrent activity against *Aedes aegypti*. As a degraded limonoid isolated from *D. dasycarpus*, the poisoned larvae of lepidoptera showed typical stomach-poisoned symptoms [24], and fraxinellone reduced the growth rates and food consumption of *T. castaneum*, *Ostrinia furnacalis* [22], tobacco budworm, *Heliothis virescens* [23] and *M. separata* [28].

It is well-known that digestive enzymes and detoxification enzymes of midgut are involved in the detoxifation or metabolism of xenobiotics [29]. In our previous paper, we reported that celangulin V (CA-V) had higher induction against two components of cytochrome P450, and inhibited GST activity in treated larvae of *M. separata* [30]. Similarly, Mukanganyama *et al*. [31] found that 2,4-dihydroxy-7-methoxy-1,4-benzoxazin-3-one (DIMBOA) decreased GST activity *in vitro* and *in vivo* by 33% and 30%, respectively. Liu *et al*. found the larval midguts of *H. virescens* significantly possessed lower α-amylase and non-specific protease activities and higher cytochromes P450 activities after treatment with fraxinellone for 24 h [23]. Based upon the above interesting results, it is suggested that the strong feeding deterrent activity and development inhibiting action of fraxinellone against *M. separata* larvae may be due to its inhibition of digestive and detoxification enzymes. The follow-up research is now being carried out in our lab.

## 3. Experimental

### 3.1. Insects and Chemicals

The larvae of Eastern armyworm, *M. separata*, diamond back moth, *P. xylostella*, black cutworm, *A. ypsilon* and *Culux pipiens pallens* were obtained from the Institute of Pesticide Science, Northwest A&F University (NWAFU). The lepidoptera larvae were reared on wheat leaves or turnip seedlings under the laboratory conditions (T = 22 °C, R.H. = 60–80%), and never contacted with insecticides. Fraxinellone (>98%) was provided by the Pharmaceutical Design & Synthesis Lab (College of Science, NWAFU) [18].

### 3.2. Larvicidal and Antifeeding Test

The bioassays of fraxinellone against the lepidoptera larvae were carried out by the traditional stomach poison method [30]. Newly molted larvae of *M. separata* (3rd and 5th instar), *A. ypsilon* (2nd instar), and *P. xylostella* (2nd instar) were selected. The larvae were firstly starved for 24 h. Fresh wheat leaf disks (0.5 × 0.5 cm) were treated with 1 μL diluted samples in acetone, and the larvae fed with them individually. Meanwhile, the control groups were fed with leaf disks treated with acetone alone. After treatment for 24 h, the residual areas of leaves were measured on plotting paper, and the AFC_50_ values were calculated from the antifeeding ratios and the corresponding concentrations [32].

The mortalities of Lepidopteran larvae were recorded until 48 h after treatment. The concentrations of fraxinellone tested against 3rd and 5th instar larvae of *M. separata* were 5.0, 7.5, 10.0, 15.0, and 20.0 mg mL^−1^, respectively. Against the 2nd instar larvae of *P. xylostella* and *A. ypsilon*, the concentrations were 0.625, 1.25, 2.5, 5.0, 10.0, and 20.0 mg mL^−1^, respectively.

The insecticidal activity of fraxinellone against mosquito larvae was tested according the method of Cheng *et al*. [33], and the final concentrations were 20.0, 40.0, 60.0, 80.0, and 200.0 μg·mL^−1^, respectively. After treatment for 24 h, once the mosquito larvae could not swim on the surface of liquid when a stimulus was given, they were deemed dead.

### 3.3. Ovicidal Test

Newly laid plump egg masses of *M. separata* were collected. The egg quantities in every egg mass were recorded, and then they were dipped into acetone solutions of fraxinellone for 3 s at concentrations of 5.0, 10.0, and 20.0 mg mL^−1^, respectively. Filter papers were used to absorb the excess liquid immediately. Then, the treated egg masses were transferred into Petri dishes (60 mm) with absorbent papers on the bottom. Fresh wheat leaves were used to keep the humidity. Every group of tests was repeated three times and the control one was only treated with acetone. Finally, all of them were moved to a rearing-room and observed every day up to about 10 days until all the eggs hatched and 2nd instar larvae emerged. The eggs were maintained in Petri dishes at 22 °C, R.H. = 60–80%. The hatch proportion of eggs (HP), the inhibition rate of egg hatching (HIR) and the development rate (DR) were calculated by the formulae:

HP (%) = the number of 1st instar larvae × 100/the number of total egg
HIR (%) = (*A*−*B*)/*A*
DR (%) = the number of 2nd instar larvae × 100/the number of 1st instar larvae
Where *A* is the HP of control, *B* is the HP of the treated larvae.

### 3.4. Development Tests

Newly molted 5th instar larvae of *M. separata* were starved for 24 h. After measuring their initial weight, the larvae were individually put into Petri dishes, where fresh leaf disks (0.5 cm × 0.5 cm) were treated with different concentrations of fraxinellone solutions at 5.0, 10.0, and 20.0 mg mL^−1^, respectively, and the procedure fowwoed was the same as for the antifeeding activity assay. After treatment for 2 h, the residual areas of wheat leaves were measured by plotting paper, and the untreated fresh leaves were added to all dishes until all the alive insects pupated and eclosed (T = 22 °C).

All the pests were then classified as follows: (1) zero grade, died within 72 h after treatment; (2) 1st grade, died in larval period after 72 h; (3) 2nd grade, died in prepupal period, or abnormal pupae appeared; (4) 3rd grade, adult emergence.

Corrected abnormal mortality (%) = (abnormal mortality of treated group − abnormal mortality of control)/(1 − abnormal mortality of control) × 100

Index of developmeng (%) = ∑ (every number of grade × related number of pest)/(the highest grade number × total number of pest) × 100

Development inhibition rate (%) = (Index of control − Index of treatment)/Index of control × 100

### 3.5. Statistical Analysis

The quantities of egg hatching and larvae growing were recorded day by day. The LC_50_ and AFC_50_ values against the four insects were calculated using EXCEL software or the DPS system.

## 4. Conclusions

In conclusion, fraxinellone, a degraded limonoid isolated from *D. dasycarpus*, exhibited strong feeding-deterrent and toxic effects against Lepidopteran larvae, including *M. separata*, *A. ypsilon* and *P. xylostella*. It also possessed high toxicity against *Culux pipiens pallens*. Besides that, after treatment with fraxinellone, the egg hatching duration and developmental period of *M. separata* were clearly prolonged. This will lay the foundation for the development of fraxinellone as a botanical insecticidal agent in organic food production.
